# Joint Associations of Physical Activity and Hypertension with the Development of Type 2 Diabetes among Urban Men and Women in Mainland China

**DOI:** 10.1371/journal.pone.0088719

**Published:** 2014-02-13

**Authors:** Fei Xu, Robert S. Ware, Lap Ah Tse, YouFa Wang, ZhiYong Wang, Xin Hong, Emily Ying Yang Chan, David W. Dunstan, Neville Owen

**Affiliations:** 1 Nanjing Municipal Center for Disease Control and Prevention, Nanjing, China; 2 School of Population Health, The University of Queensland, Brisbane, Australia; 3 JC School of Public Health and Primary Care, The Chinese University of Hong Kong, Hong Kong, China; 4 Department of International Health, Johns Hopkins Bloomberg School of Public Health, University, Baltimore, Maryland, United States of America; 5 Baker IDI Heart and Diabetes Institute, Melbourne, Australia; McGill University, Canada

## Abstract

**Background:**

Physical activity (PA) and hypertension (HTN) are important influences on the development of type 2 diabetes (T2D). However, the joint impact of PA and HTN on T2D development is unknown.

**Methods:**

Two community-based prospective cohort studies, with the same protocols, instruments and questionnaires, were conducted among adults in urban areas of Nanjing, China, during 2004–2007 and 2007–2010. T2D was defined using World Health Organization criteria based on physicians' diagnosis and fasting blood glucose concentration. PA level (sufficient/insufficient) and blood pressure status (hypertensive/normotensive) were assessed at baseline and the third year of follow-up. We pooled and analyzed data from these two studies.

**Results:**

Among 4550 participants aged 35 years or older, the three-year cumulative incidence of T2D was 5.1%. After adjusting for potential confounders, participants with sufficient PA were less likely to develop T2D than those with insufficient PA (OR = 0.43, 95%CI = 0.27, 0.68) and those who were normotensive were less likely to develop T2D than those who were hypertensive (OR = 0.39, 95%CI = 0.29, 0.51). Compared to participants with insufficient PA and who were hypertensive, those with sufficient PA and hypertension were at lower risk of developing T2D (OR = 0.36, 95%CI = 0.19, 0.69), as were those with insufficient PA who were normotensive (OR = 0.37, 95%CI = 0.28, 0.50) and those with sufficient PA who were normotensive (OR = 0.19, 95%CI = 0.10, 0.37).

**Conclusions:**

Insufficient PA was found to be associated with the development of T2D among adults with and without hypertension. These findings support a role for promoting higher physical activity levels to lower T2D risk in both hypertensive and non-hypertensive individuals.

## Introduction

Hypertension (HTN) and type 2 diabetes (T2D) are two common chronic conditions that have significant impacts on health care systems in both the developed and developing world [1]–[6]. In China, the prevalence of hypertension and diabetes among adults in 2008 was estimated as 26.9% and 9.7%, respectively [5], [6]. China is estimated to contain one in five of the worldwide population of adults with hypertension and one in three with diabetes [7], [8]. The Chinese prevalence estimates are similar to those for the USA in 2004, which were 29.3% and 7.8% [3], [4]. Two large prospective cohort studies in the USA showed that the development of T2D was about 2.5 times more likely in hypertensive persons than in their normotensive counterparts [9], [10]. Hypertension and T2D, as major components of the metabolic syndrome, often co-exist, and lead to a worsening in cardiovascular prognosis and greater burden than do each of the conditions individually [9], [10]. Effective strategies to prevent T2D in people with hypertension are urgently needed, especially in countries with a high population prevalence of hypertension.

It is widely acknowledged that physical inactivity contributes to an elevated risk of developing T2D [11], [12], [13]. Consequently, current public health guidelines relating to PA emphasize the weekly participation in moderate-intensity physical activity for at least 30 minutes on at least 5 days, and/or vigorous physical activity for at least 20 minutes on at least 3 days as sufficient for preventive benefits [13].

Although physical inactivity and hypertension have been identified as two independent risk factors for T2D individually, the joint associations of physical inactivity and hypertension with the risk of developing T2D are unknown. We hypothesize that physical inactivity and hypertension may exert additive impacts on the development of T2D relative to each of them separately. The aim of this study was to examine the joint association of physical inactivity and hypertension on the risk of developing T2D, using pooled data from two cohorts of adults living in a large regional city in Mainland China.

## Methods

### Study Design and Sample

The data used in this report were pooled from two three-year community-based prospective cohort studies conducted in urban areas of Nanjing, Mainland China, with the original purpose for investigating the influence of lifestyle on the risk of developing T2D. These two cohort studies shared the same methodology and sampling approach which has been described in detail elsewhere [14]. Briefly, there was a total of seven urban communities included in this pooled study, with three in the first cohort (study dating from July 2004 to July 2007) and four in the second cohort. (July 2007 to July 2010). Adults were eligible to participate if they were regular residents in the selected community, and aged 35 years or older. People were excluded if they had been previously diagnosed with cancer or diabetes. The written informed consent was obtained from each participant prior to the survey. Both cohort studies were separately approved by the academic and ethical committee of Nanjing Municipal Center for Disease Control and Prevention (Nanjing CDC).

### Outcome Variable

Apart from questionnaire and measurement on body weight and height, we tested each participant's blood glucose at the baseline survey and then excluded those with T2D confirmed by endocrinologist from the baseline. We re-tested each cohort member's blood glucose level at study end to identify the new occurrence of T2D during the three-year follow-up period, while the diagnosis of T2D was made by the endocrinologists according to the fasting venous blood glucose concentration (tested with Glucose Oxidase Method) and the presentation of clinical symptoms relevant to diabetes based on the 1999 World Health Organization criteria [15]. In addition, we provided each participant with free fasting blood glucose tests in a designated local hospital during the 3-year follow-up.

### Exposure Variables

The information on self-reported PA in the past week was collected using the validated Chinese short-version of the International Physical Activity Questionnaire (IPAQ-CHN) [16]. The total PA time was calculated as the sum of the time spent in moderate-intensity PA plus double the time spent in vigorous-intensity PA according to the recommendations by U.S. Department of Health and Human Services [13]. Participants were classified as having either sufficient (total PA time ≥150 minutes/week) or insufficient (total PA time <150 minutes/week) PA [13].

Participants were recorded as being hypertensive if they had been diagnosed by a registered hospital physician based on their blood pressure status and clinical symptoms [17]. All participants prescribed antihypertensive drugs were asked to show their personal medical records to confirm the previous diagnosis with hypertension and then classified as being hypertensive.

We categorized participants according to their PA and blood pressure status into four subgroups: (1) insufficient PA and hypertension (reference group, ‘the highest risk group’), (2) sufficient PA and hypertension, (3) insufficient PA and normotension, and (4) sufficient PA and normotension (‘the lowest risk group’).

Each participant's height and weight were measured twice at each visit following standardized procedures, and the mean of the two readings was adopted for the calculation of BMI [weight (kg)/height (m^2^)]. Following Chinese recommendations, participants were classified as overweight (24≤ BMI <28), and obesity (BMI ≥28) [18]. In addition, each participant's information on smoking, alcohol drinking, television viewing (a typical type and widely used indicator of sedentary behavior), consumption of meat and vegetables, and family history of T2D were also collected. A participant was classified as a current smoker if he or she had smoked at least one cigarette per day continuously for at least the past year, or had smoked a total of at least eighteen packs in the past year, and as an alcohol drinker if he or she drank alcohol an average of two or more times per week for at least one year. We adopted a validated Frequency Food Questionnaire (FFQ) to gather information on dietary consumption including meat and vegetables in a quantitative scale, but the collection of fruit was based on the frequency of usage [19]. A participant who reported having at least one parent diagnosed with T2D was recorded as having a positive family history.

### Statistical Analysis

Descriptive statistics and three-year cumulative incidence rates of T2D are reported. We investigated the effect of PA and hypertension status on T2D using univariate and multivariate logistic regression models to obtain the odds ratio (OR) and the 95% confidence interval (95% CI). We also included a PA/HTN interaction term in the multivariate logistic regression models to examine the multiplicative interaction between PA and HTN on the risk of T2D. In addition, multivariate logistic regression analyses were further performed to estimate the effect of PA and HTN in these three pairs (sufficient PA and hypertension, insufficient PA and normotension, and sufficient PA and normotension) by using the pair “insufficient PA and hypertension” as the reference group. Classical influential factors that were regarded as the potential confounding factors were age, gender, educational attainment, BMI category, family history of diabetes, cigarette smoking, alcohol drinking, TV viewing, vegetable intake and meat consumption. Data analyses were conducted using SPSS 21.0 (IBM Corp, Armonk, NY, USA).

## Results

Over both three-year studies, 6,309 were eligible to participate, 5,659 (89.7%) participated at baseline and 4,550 (80.4% of baseline) completed the follow-up survey. Participation and follow-up percentages for the 2004-07 and 2007-10 cohorts were 91.8% (2677/2915) and 81.3% (2177/2677), and 87.9% (2982/3394) and 79.6% (2373/2982) respectively. Overall, participants followed-up were similar to those who were not in terms of age (mean [SD]  = 51.8[10.6] years *vs.* 51.1 [10.2] years) and gender (42.9% men *vs.* 43.2% men).

Selected baseline characteristics of participants according to PA/hypertension category and the diabetes status at the three-year of follow-up are shown in Table 1. The mean [SD] age for participants was 51.8[10.6], while 10.9% of participants were classified as obese, 32.2% identified having hypertension and 16.5% engaging sufficient PA.

**Table 1:** The baseline characteristics according to physical activity/hypertension category and the follow-up diabetic status among urban participants in Nanjing City, China*.

Characteristics	Total	PA/hypertension categories at baseline				Presence/absence of diabetes at 3-year follow-up§	
		Insufficient PA and hypertension	Sufficient PA and hypertension	Insufficient PA and normotension	Sufficient PA and normotension	Diabetes	No diabetes
No. of participants	4550	1200	263	2597	490	232	4318
Mean age (SD)	51.8 (10.6)	55.5 (9.8)	55.6 (10.1)	50.0 (10.6)	50.5 (10.4)^‡^	55.4 (9.4)§	51.6 (10.6)
Gender (% Men)	42.9	43.9	44.5	41.5	46.5	47.8	42.6
Educational level (13+yrs) (%)	12.4	8.8	10.6	13.8	14.5^‡^	6.9^‡^	12.6
Sufficient Physical Activity (≥150 m/wk; %)	16.5	N/A	N/A	N/A	N/A	9.1§	17.0
Hypertension (%)	32.2	N/A	N/A	N/A	N/A	56.9§	30.8
Viewing TV time < 1 hr/day (%)	9.2	8.8	9.5	9.4	9.6	8.6	9.3
Current smoker (%)	24.4	24.2	25.1	24.0	26.7	26.3	24.3
Alcohol drinker (%)	12.8	12.3	15.2	12.2	15.9	12.9	12.8
Mean (SD) of vegetables intake (g/day)	332.4 (218.6)	317.0 (168.4)	368.1 (231.9)	321.8 (176.3)	407.0 (417.0)^‡^	299.4 (164.0)^‡^	334.2 (221.1)
Mean (SD) of meat intake (g/day)	65.8 (54.0)	62.6 (53.1)	60.0 (49.2)	68.6 (55.4)	62.3 (50.2)†	64.8 (45.0)	65.9 (54.4)
BMI ≥28 (kg/m^2^) (%)	10.9	14.3	21.7	8.9	7.8^‡^	11.6	10.9
Positive Family history of diabetes (%)	9.4	9.3	16.0	7.3	17.3^‡^	19.8§	8.8

* Continuous variables were presented as Means ± SD, while categorical variables, percentages.

§ Diabetes was diagnosed based on venous blood glucose test plus clinical symptoms by registered physicians according to the 1999 World Health Organization diagnostic criterion.

Sufficient PA refers to total moderate PA time ≥ 150 minutes/week.

† *p*<0.05, ‡ *p*<0.01, the difference between men and women, and between participants with different PA/hypertension categories was significant.

N/A: Not applicable.

The three-year cumulative incidence of T2D was 5.1% (95%CI = 4.5, 5.8). There were no statistically-significant differences by gender, time spent in viewing TV, and intake of meat and percentage of obesity between those who developed and did not develop T2D during the follow-up period. However, compared to their counterparts who did not develop T2D, those who developed T2D tended to be older, have lower educational attainment and consume less vegetables.

Table 2 presents the separate association of PA and hypertension with subsequent T2D. The cumulative incidence of T2D was 9.0% (95%CI = 7.6, 10.6) among those with hypertension and 3.2% (95%CI = 2.7, 3.9) for those with normal blood pressure, while for participants with insufficient PA it was 5.6% (95%CI = 4.9, 6.4) and for those with sufficient PA it was 2.8% (95%CI = 1.8, 4.3). After adjustment for potential confounders, the odds of participants with sufficient physical activity being newly diagnosed with T2D were approximately half those of participants with insufficient physical activity being newly diagnosed (OR = 0.43, 95%CI = 0.27, 0.68), while those without hypertension were significantly less likely to have subsequent T2D (OR = 0.39; 95%CI = 0.29, 0.51) compared to their counterparts with hypertension. The interactions between PA and hypertension (P = 0.39), gender and PA (P = 0.72), and gender and hypertension (P = 0.59), on the risk of developing T2D were not statistically significant.

**Table 2:** The separate association of physical activity and hypertension with subsequent diabetes at three-year follow-up among overall sample population in urban areas of Nanjing, China*.

Exposure variables	N of participants	Participants who developed diabetes†		
		% (n)	OR (95%CI)	
			Model 1‡	Model 2§
**Physical Activity** ^∥^				
Insufficient	3797	5.6 (211)	1.00	1.00
Sufficient	753	2.8 (21)	0.49 (0.31, 0.77)	0.43 (0. 27, 0.68)
**Hypertension**				
Yes	1463	9.0 (132)	1.00	1.00
No	3087	3.2 (100)	0.34 (0.26, 0.44)	0.39 (0.29, 0.51)

* Odds ratio (OR) and 95% CIs were used to present the influence of baseline moderate physical activity and hypertension on subsequent diabetes, separately, among overall sample population.

† Diabetes was diagnosed based on venous blood glucose test plus clinical symptoms by registered physicians according to the 1999 World Health Organization diagnostic criterion. Participants with normal blood glucose values were treated as the reference.

‡ Model 1: univariate logistic regression model.

§ Model 2: multivariate logistic regression model with adjustment for age, gender, educational attainment, family history, PA/hypertension,body weight status, cigarette smoking, alcohol drinking, TV viewing, vegetables intake and meat intake.

∥ Physical activity was categorized into ‘Sufficient’ and ‘Insufficient’ based on the recommendation (at least 150 minutes per week) for adults'

The joint influence of PA and hypertension on the risk of developing T2D is displayed in Table 3. Compared to the reference group with both insufficient PA and hypertension, participants with sufficient PA and hypertension were at lower risk of developing T2D (OR = 0.39, 95%CI = 0.21, 0.73), and those with insufficient PA and normal blood pressure were also less likely to develop T2D (OR = 0.32, 95%CI = 0.24, 0.42), while those recording sufficient PA and normal blood pressure were least likely to develop T2D (OR = 0.19, 95%CI = 0. 10, 0.36). These estimates were unaffected by adjustment for potentially confounding variables. When the data were analyzed in men and women separately, similar graduated associations were found.

**Table 3:** The joint influence of physical activity and hypertension on the risk of developing diabetes at three-year follow-up among overall sample population in urban areas of Nanjing, China*.

Exposure variables		N of participants	Participants who developed diabetes†		
			% (n)	OR (95%CI)	
				Model 1‡	Model 2§
Physical Activity^∥^	Hypertension				
**Overall**					
Insufficient	Yes	1200	10.1 (121)	1.00	1.00
Sufficient	Yes	263	4.2 (11)	0.39 (0.21, 0.73)	0.36 (0.19, 0.69)
Insufficient	No	2597	3.5 (90)	0.32 (0.24, 0.42)	0.37 (0.28, 0.50)
Sufficient	No	490	2.0 (10)	0.19 (0.10, 0.36)	0.19 (0. 10, 0.37)

* Odds ratio (OR) and 95% CIs were used to present combined influence of baseline moderate physical activity and hypertension on subsequent diabetes.

† Diabetes was diagnosed based on venous blood glucose test plus clinical symptoms by registered physicians according to the 1999 World Health Organization diagnostic criterion. Participants with normal blood glucose values were treated as the reference.

‡ Model 1: univariate logistic regression model.

§ Model 2: multivariate logistic regression model with adjustment for age, gender, educational attainment, family history of diabetes, body weight status, cigarette smoking, alcohol drinking, TV viewing, vegetables intake and meat intake.

∥ Physical activity was categorized into ‘Sufficient’ and ‘Insufficient’ based on the recommendation (at least 150 minutes per week) for adults'.

## Discussion

This pooled analysis of data from 4,550 urban Chinese adults revealed that those with sufficient PA had a significantly lower risk of developing T2D than those with insufficient PA, and that those with normal blood pressure were significantly less likely to develop T2D than their hypertensive counterparts. We observed that the odds of developing T2D decreased across the combined PA/hypertension groups from insufficient-PA/hypertension, sufficient-PA/hypertension, insufficient-PA/normotension, to sufficient-PA/normotension – with the latter having the lowest risk. Moreover, normotensive participants with insufficient PA were at similar risk of developing T2D as those who were hypertensive and had sufficient PA; and both were at significantly lower risk of developing T2D than their hypertensive counterparts with insufficient PA. Having sufficient PA was associated with a 57% reduction in the odds of developing T2D relative to those recording insufficient PA. Normotensive participants demonstrated a 64% reduction in the odds of subsequent T2D compared to hypertensive participants, while the odds of normotensive participants who recorded sufficient PA developing T2D were 81% lower than participants with insufficient PA and hypertension. These findings suggest there may be joint protective benefit from sufficient PA and normotension compared to insufficient PA or hypertension individually in this population.

To the best of our knowledge, this is the first community-based prospective cohort study examining the combined influence of PA and hypertension on the risk of developing T2D. The findings may be of significance for populations where obesity and its consequent complications- particularly T2D- are becoming major public health problems [5], [6], [8]. It emphasizes the particular importance of physical activity participation among those for whom hypertension places them at increased risk of developing T2D.

In the present study, hypertension was shown to be an independent risk factor for T2D, consistent with previous studies [9], [10]. The association between hypertension and type-2 diabetes is complex and may involve several mechanisms. In addition to the widely accepted role of aging and increased body weight, hypertension may contribute to the onset of diabetes through the following pathways: (1) reducing the delivery of insulin and glucose by decreasing blood flow of skeletal muscle, the major uptaker of body glucose, due to increased vasoconstriction and vascular rarefaction; (2) diminishing insulin-sensitivity of skeletal muscle slow-twitch fibers; and (3) decreasing insulin postreceptor signaling through PI3K-Akt pathway. [20]. Hypertension and T2D share many of the same contributing behavioral and biological risk factors, such as obesity, physical inactivity, high-dense-energy food and adverse levels of C-reactive protein, TNF-a and IL-6 [21], [22]. Hypertension and T2D may have an additive effect on each other, with one condition both facilitating the onset, and worsening manifestations, of the other [23].

Previous epidemiological studies have demonstrated that PA contributes to the reduction in the risk of developing T2D [11], [12]. Hypertension is a strong and independent risk factor for T2D [13]–[18], [24]. Thus, it is possible that the impact of physical activity on subsequent T2D might be different for those with and without hypertension.

Strengths of this study include its large community-based sample size, prospective design, high recruitment and follow-up percentages, assessment of PA using a widely used questionnaire, validation of hypertension and T2D using medical records, and analytic adjustment for potentially confounding variables. Our findings have emerged through using data derived from two separate community-based cohorts which were designed and administered by the same research team using the same study protocol and methodology (including sampling approaches, instruments, and questionnaires) [14]. Only urban participants were recruited in order to ensure comparable socio-economic status of participants between study cohorts, while three-year interval between two studies minimized the differences in participants' lifestyle and behavioral pattern.

Limitations of the study are that the three year follow-up period is relatively short when investigating a chronic condition such as T2D. Although the overall sample size was large, there were only 753 participants meeting the level of sufficient PA in the sample population, which resulted in a small number of participants with newly-identified T2D in the sufficient-PA/hypertension and the sufficient-PA/normotension subgroups over the three-year follow-up period.

In conclusion, this study provides evidence to support the hypothesis that physical inactivity in combination with hypertension may additively increase the risk of developing T2D. Because hypertension is an independent risk factor for T2D, and shares common modifiable risk factors with T2D, those with hypertension may stand to gain significant benefit in reducing their risk of developing T2D through regular physical activity participation.
